# Chronic Pain: The Need and Hope for Opioid Alternatives

**DOI:** 10.1016/j.ebiom.2016.03.011

**Published:** 2016-03-19

On January 11, 2016, a Phase 1 trial in France of the investigational drug BIA 10-2474 was abruptly halted when one of the six dosed subjects fell into a coma after experiencing symptoms resembling a stroke. The subject died several days later and four others were left with what doctors suspect may be lasting neurological damage. What precipitated this tragedy is still unclear. As an inhibitor of fatty acid amide hydrolase, a critical enzyme in the biosynthesis of endocannabinoids, it was thought that BIA 10-2474 could target aberrant neurotransmission in disorders ranging from anxiety and Parkinson's disease to chronic pain. While the failure of the BIA 10-2474 trial should not be downplayed, it does at least herald the critical need for further investigation into new ways of treating nervous system disorders — including chronic pain, whose current therapies include those that may themselves be perilous.

Chronic pain is generally defined as that which lasts between three and six months after the acute symptoms should have subsided. It can arise from myriad conditions including arthritis, diabetic neuropathy, cancer, traumatic injury, certain viral infections, and many other diseases that lead to persistent inflammation or nerve dysfunction. Roughly 25% of people worldwide will at some point suffer from chronic pain. Consequently, the WHO estimates chronic pain to be one of the leading causes of Years Lost to Disability globally. In the US alone, where up to 12 million people have chronic pain, this equates to an economic burden of nearly $635 billion per year.

Opioids are widely used as first line drugs for both acute and chronic moderate to severe pain. While opioids often produce unmatched analgesic effects, at sufficient doses or with prolonged use they can trigger psychoactive effects, tolerance, and addiction. Prescription opioid abuse has exploded in developed nations over the last decade. In the US, the epidemic has grown to such proportions that the Centers for Disease Control estimates over 19,000 people died from overdose of prescription opioids in 2014, a cause of death whose rate now exceeds that of motor vehicle accidents. These troubling statistics led the US Food and Drug Administration in February, 2016 to largely amend its stance on opioids. Among other policy statements was a call to action to develop novel therapies with reduced potential for misuse.

Given that opioids are the only currently-available means of relief from chronic pain for many patients, one branch of research focusses on ways to limit their use at the high doses necessary to elicit harmful effects. These efforts include drug formulations that confer delayed release and technologies such as implantable devices that dispense precise doses to provide only analgesia. Other research is aimed at developing opioids with fewer adverse effects. Of the mu, delta, and kappa subtypes of opioid receptors, most analgesia is thought to derive from mu activation. Recent studies in pre-clinical pain models suggest that a synthetic opioid derivative, UMB 425, which both agonizes mu and antagonizes delta receptors, provides analgesia without the development of tolerance. Another compound, MMG22, both agonizes mu receptors for analgesia and antagonizes metabotropic glutamate receptors thought to mediate addiction. Other efforts are aimed at developing molecules with selective binding affinities for different opioid receptor subtypes to reduce the risk of dependence and overdose, including the mu-biased agonist oliceridine that recently won FDA Breakthrough Drug approval and is in Phase 3 development.

Basic neuroscience findings on pain have led to targets not related to opioid receptor modulation. The γ-aminobutyric acid (GABA) receptor agonists gabapentin and pregabalin have been approved for various chronic pain syndromes for several years, as have the serotonin-norepinephrine reuptake inhibitors (SNRIs) duloxetine and minacipran. In the pre-clinical pipeline, researchers are exploring a range of ion channels, receptors, and pathways to modulate pain. Sodium and calcium channels that are selectively expressed in sensory neurons are an attractive target as they could provide analgesia similar to traditional anesthetics without central depressant effects. Compounds that block capsaicin receptors and transient receptor potential (TRP) channels on nociceptive neurons or purinergic and TLR4 receptors on glial cells have also been shown to confer analgesia. An angiotensin Type II receptor antagonist, EMA401, recently showed efficacy in a Phase 2 trial for post-herpetic neuralgia. Several groups are also investigating anti-nerve growth factor (NGF) antibodies, with clinical trials underway using tanezumab for the treatment of osteoarthritis and cancer pain.

Cannabis can be an effective treatment for some chronic pain sufferers and is available by prescription for in some jurisdictions. As with opioids, though, there are concerns about the psychoactive effects of cannabis and its potential for misuse. Therefore, synthetic molecules that selectively interact with only therapeutic targets are being investigated. Among them, the cannabinoid-derivatives nabilone and dronabinol are approved in many regions as adjunctive treatments for certain chronic pain conditions. Modulators of the body's biosynthetic pathways of endocannabinoids — including inhibitors of fatty acid amide hydrolase like BIA 10-2474 — are being explored by many groups. Despite the recent tragedy related to BIA 10-2474, the endocannabinoid system is still a promising pain target.

Many questions remain unanswered about the BIA 10-2474 trial in France. With the investigation still ongoing it would be irresponsible to speculate on the specifics of this trial's failure. Along with ensuring trials are designed and conducted with patient safety at the fore, we must also remain diligent in weighing the rewards and risks of translating pre-clinical findings to the clinic. Currently, with opioids the standard of care for pain management, doctors and patients must weigh the rewards and risks of such treatment: analgesia versus potential misuse. Viable alternatives to opioids remain a much-needed reward for sufferers of chronic pain.

-*EBioMedicine*

